# Meniscus progenitor cells combined with joint lavage promote meniscus regeneration and cartilage protection in rat models

**DOI:** 10.3389/fbioe.2025.1724656

**Published:** 2026-01-21

**Authors:** Shu-Yang Guo, Jia-Hao Zhu, Wan-Ting Yan, Xiao-Jia Huang, Wen-Nan Xu, Jing-Song Wang, Zheng-Zheng Zhang

**Affiliations:** Department of Sports Medicine, Sun Yat-sen Memorial Hospital, Sun Yat-sen University, Guangzhou, China

**Keywords:** cartilage, cell therapy, IL-1 beta, meniscus, meniscus progenitor cells, rat models, tissue regeneration

## Abstract

The meniscus plays a crucial role in knee joint function, yet its limited intrinsic regenerative capacity, particularly within the avascular region, makes meniscal injuries a major therapeutic challenge. Meniscus progenitor cells (MPCs) have shown potential to promote meniscal regeneration, but their efficacy may be compromised in the inflammatory microenvironment. Joint lavage, a simple and clinically applied procedure that reduces intra-articular inflammation, may enhance the outcomes of cell-based therapies. Here, we investigated the therapeutic efficacy of combining joint lavage with MPC transplantation in rat partial meniscectomy models and further examined the effects of interleukin-1β (IL-1β), a key inflammatory mediator, on MPC function *in vitro*. Lavage significantly reduced intra-articular levels of multiple inflammatory cytokines, including IL-1β, which otherwise impaired MPC migration, proliferation, chondrogenic differentiation, and gene expression. *In vivo*, combined lavage and MPC therapy promoted more complete meniscal regeneration, enhanced type II collagen deposition, preserved cartilage integrity, and improved biomechanical properties compared with either treatment alone. These findings demonstrate that preconditioning the joint microenvironment through lavage markedly augments the regenerative potential of MPCs, providing a simple, low-cost, and clinically feasible strategy to improve meniscus and cartilage repair under inflammatory conditions.

## Introduction

The meniscus, a critical component of the knee joint, has limited self-repair capacity, particularly in the avascular zone (Arnoczky and Warren, 1982). As a result, injuries like meniscal tears present a significant clinical challenge (Gong et al., 2023; Anderson et al., 2011; Trivedi et al., 2021). Currently, partial meniscectomy remains one of the most common clinical treatments for meniscal injuries. However, evidence shows that while partial meniscectomy alleviates symptoms and restores function in the short term, the resultant meniscal defect increases the risk of post-traumatic osteoarthritis (PTOA) and cartilage degeneration (Longo et al., 2019; Rangger et al., 1995; Scotti et al., 2013; Evers et al., 2022).

Stem cell-based strategies for tissue repair and regeneration are currently emerging as promising approaches for tissue repair and regeneration. In particular, Mesenchymal stem cells (MSCs) have shown potential in promoting meniscal regeneration and cartilage repair (Dai et al., 2021; Zhou et al., 2022). However, the inflammatory environment in joint cavity accelerates MSCs senescence, suppresses chondrogenic potential, as well as impairs adhesion and migration capabilities (Heldens et al., 2012; Reesink et al., 2017; Peng et al., 2022).

In this inflammatory environment, cytokines serve essential regulatory functions (Punzi et al., 2016). Pro-inflammatory cytokines have been shown to inhibit the chondrogenic differentiation of MSCs by inducing apoptosis and extracellular matrix degradation, limiting their chondroprotective efficacy (Pattappa et al., 2019; Buhrmann et al., 2010; Wehling et al., 2009). Among these, interleukin-1β (IL-1β) plays an essential role in PTOA pathogenesis. It acts both as an initiator and perpetuator of intra-articular inflammation and directly leads to cartilage degeneration (Kapoor et al., 2011).

Knee joint lavage, a simple commonly used clinical procedure, which could effectively eliminate pro-inflammatory cytokines such as IL-1β and TNF-α for reducing joint inflammation or wiping out infection (Li et al., 2023; Fu et al., 2009; Ravaud et al., 1999). Yet, studies report poor long-term outcomes following lavage, with limited improvements in pain and function. Therefore, joint lavage require other synergetic treatment (Avouac et al., 2010; Reichenbach et al., 2010).

Meniscus progenitor cells (MPCs) have recently emerged as a promising source for meniscus regenerative therapies (Yan et al., 2024a). Compared with bone marrow-derived MSCs (BMSCs), MPCs demonstrate superior chondrogenic differentiation and higher type II collagen expression *in vitro*. Animal studies further support their enhanced meniscal healing capacity over BMSCs (Gui et al., 2015; Ding and Huang, 2015). While previous animal studies have confirmed regenerative potential of MPCs (He et al., 2019; Shen et al., 2014; Shen et al., 2013), these typically involved repeated injections within 2 weeks after injury, which does not fully reflect the clinical situation. Importantly, the inflammatory environment at this stage—particularly elevated cytokines such as IL-1β—may compromise MPC function, representing a barrier to their clinical translation.

Building upon these insights, we hypothesize that knee joint lavage can create a more favorable intra-articular environment for MPCs injection by clearing key inflammatory factors—predominantly IL-1β—and improving the intra-articular milieu. Accordingly, this study has two aims: 1. *In vitro*: to investigate the specific effects of IL-1β on the biological properties of MPCs; and 2. *In vivo*: to evaluate the therapeutic efficacy of combining joint lavage with MPCs injection for meniscus regeneration and cartilage preservation. Collectively, these findings are expected to provide a theoretical framework for improving the clinical translation of MPCs therapies.

## Materials and methods

### MPCs isolation

All animal experiments were conducted following approval from the Institutional Animal Care and Use Committee (IACUC) of Sun Yat-sen University (2024-002280). MPCs were isolated using differential adhesion to fibronectin (Yan et al., 2024b; Wang et al., 2022; Korpershoek et al., 2021). Briefly, 6-well plates were coated overnight at 4 °C with a 10 μg/mL solution of fibronectin (Sigma-Aldrich, United Kingdom) in calcium- and magnesium-enriched phosphate-buffered saline (PBS). The medial and lateral meniscus were harvested from the knees of 10 male Sprague-Dawley rats (400 ± 20 g) following euthanasia. Meniscal tissues were minced into 1 mm^3^ fragments and subsequently digested with pronase (70 U/mL for 1 h at 37 °C) followed by collagenase type II (245 U/mL for 12 h at 37 °C; Worthington, USA). The isolated cells were counted using a hemocytometer and seeded at a low density of 2 × 10^3^ cells/mL (200 cells/cm^2^) onto fibronectin-coated plates. Non-adherent cells were discarded after a 20-min incubation at 37 °C with 20% O_2_ and 5% CO_2_. Adherent cells were then cultured in DMEM supplemented with 10% fetal bovine serum (FBS) and 1% penicillin/streptomycin (P/S). MPC colonies (>32 cells/colony) were identified on day 10, expanded using 0.25% trypsin/EDTA (Gibco-Thermo Fisher Scientific, USA).

Of the 10 donor rats, MPCs from three rats were used for *in vitro* experiments, while MPCs from the remaining seven rats served as cell sources for *in vivo* transplantation. MPCs from each donor rat were expanded independently and subsequently administered to approximately five recipient rats. MPCs at passages 1–4 were used for both the *in vitro* assays and the intra-articular injections.

### Treatment of cells with IL-1β

Based on previous studies identifying IL-1β as a pro-inflammatory cytokine in the inflammatory environment (He et al., 2020; Wojdasiewicz et al., 2014; Dinarello, 2011), cultured MPCs were co-incubated with 10 ng/mL IL-1β for 24 h to establish an *in vitro* inflammatory microenvironment. Control MPCs were maintained under standard culture conditions.

### Cell viability assessment

Cell viability was assessed using a live/dead double-staining kit (Calcein-AM/PI). Briefly, untreated and IL-1β-treated MPCs were seeded in 6-well plates (5 × 10^4^ cells/well) and cultured under experimental conditions. After treatment, cells were incubated with 2 μM Calcein-AM (green fluorescence for live cells) and 1 μg/mL propidium iodide (PI, red fluorescence for dead cells) in PBS for 15–20 min at 37 °C. After three washes with PBS, fluorescent images were captured using an inverted fluorescence microscope (Olympus, IX73; Japan).

### Cell counting Kit-8 (CCK-8) assay

Untreated and IL-1β-treated MPCs were seeded in 96-well plates at a density of 2 × 10^3^ cells/well and maintained in DMEM containing 10% FBS and 1% P/S. Cell viability was assessed on days 1, 3, and 5 by adding 10 μL of CCK-8 reagent (Dojindo, Japan) per well. After 2 h of incubation, absorbance was measured at 450 nm using a microplate reader.

### Colony-forming unit (CFU) assay

Untreated and IL-1β-treated MPCs were seeded in 6-well plates at a density of 300 cells/well and cultured in DMEM containing 10% FBS and 1% P/S with medium replacement every 3 days. After 14 days of culture, cells were washed once with PBS. Colonies were then fixed with 4% paraformaldehyde (PFA) for 20 min and stained with 1% crystal violet for 30 min. Colony numbers in each group were quantified for comparison.

### Cell migration assessment

Untreated and IL-1β-treated MPCs were seeded in 6-well plates at a density of 5 × 10^5^ cells/well and cultured until 100% confluence. Linear wounds were created using a sterile 1,000 μL pipette tip, followed by PBS washing and replacement with FBS-free culture medium. Wound areas were imaged at 0, 12, and 24 h using an inverted microscope (Olympus, IX73; Japan). Migration rates were quantified by calculating the ratio of the migrated distance to the initial wound width.

### Flow cytometry analysis

Untreated and IL-1β-treated MPCs (5 × 10^5^ cells) were resuspended in PBS containing 5% (w/v) bovine serum albumin (BSA; BioFroxx, Germany) and incubated at 4 °C for 30 min to block nonspecific binding. Cells were then stained with fluorescence-conjugated antibodies against stem cell and progenitor cell markers (CD90, CD45, CD44, CD34; all 1:100 dilution) (Dominici et al., 2006) for 30 min at 4 °C. Antibody-stained cells were analyzed using a BD FACSVerse™ flow cytometer (BD Biosciences, USA), and data were analyzed with FlowJo software (v10.8.1).

### Multilineage differentiation potentials

To assess the osteogenic potential of the cells, untreated and IL-1β-treated MPCs (1.5 × 10^5^ cells/well) were cultured in 6-well plates with osteogenic induction medium (Procell, PD-014). The induction medium was replaced every 3 days. After 21 days of differentiation, mineralized matrix deposition was assessed by staining with 0.2% Alizarin Red S solution (Procell) for 30 min.

To assess the adipogenic potential of the cells, untreated and IL-1β-treated MPCs (1.5 × 10^5^ cells/well) were cultured in 6-well plates with adipogenesis induction medium (Procell, PD-016). The induction medium was replaced every 3 days. After 21 days of differentiation, lipid droplets were visualized by staining with Oil Red O solution (Procell) for 30 min.

To assess the chondrogenic potential of the cells, untreated and IL-1β-treated MPCs (4 × 10^5^ cells) were pelleted by centrifugation (250 × g for 5 min) and cultured in chondrogenic differentiation medium (Procell, PD-015) according to the manufacturer’s instructions. After 28 days of differentiation, pellets were paraffin-embedded, sectioned at 5 μm thickness, deparaffinized, and stained with Alcian Blue (Procell) for 30 min.

### Quantitative real-time reverse transcription polymerase chain reaction

Quantitative polymerase chain reaction (qPCR) was conducted to evaluate the expression of inflammatory and chondrogenesis-related genes in untreated and IL-1β-treated MPCs. Total RNA was isolated using a purification kit (EZBioscience, USA), and RNA concentration and purity were verified using a NanoDrop 2000 (A260/A280 ratio >1.8; Thermo Fisher, USA). Reverse transcription was performed with 1 μg RNA using the EZscript RT Reagent Kit (EZBioscience, USA) under standard conditions (37 °C for 15 min, 85 °C for 5 s). qPCR amplification was carried out in 96-well plates using SYBR Green qPCR Master Mix (EZBioscience, USA) on a QuantStudio 3 Real-Time PCR System (Applied Biosystems, USA). Primer sequences are provided in Supplementary Table S1. Thermal cycling conditions included an initial denaturation at 95 °C for 30 s, followed by 40 cycles of 95 °C for 5 s (denaturation) and 60 °C for 30 s (annealing/extension). Relative mRNA expression was calculated using the 2^−ΔΔCT^ method and analyzed with GraphPad Prism (v9.3.0; GraphPad Software, USA).

### Animal models and surgical procedures

All animal experiments were approved by the Institutional Animal Care and Use Committee (IACUC) of Sun Yat-sen University (2024-002280) and conducted in accordance with institutional and national guidelines for the care and use of laboratory animals. A total of 85 healthy male Sprague–Dawley rats (10 weeks old) were randomly assigned to five groups: sham group (Sham), partial medial meniscectomy group (Untreated), joint lavage group (Lavage; lavage performed 4 weeks after surgery), meniscus progenitor cell injection group (MPCs), and combined lavage and MPC injection group (Lavage + MPCs). For each group, four rats were evaluated at each time point (n = 4), except at week 12, where five rats were included to allow for an additional medial meniscus specimen for biomechanical properties evaluation.

Anesthesia was induced with 5% isoflurane in oxygen at a flow rate of 2 L/min and maintained with 1.5%–2% isoflurane at a flow rate of 1.5 L/min using a small animal gas anesthesia system. Adequate anesthetic depth was confirmed by the absence of the pedal withdrawal reflex. Partial medial meniscectomy was performed on the right knee under isoflurane inhalation anesthesia. The surgical area was shaved and aseptically prepared. A medial parapatellar incision (approximately 1 cm) was made from the femoral medial condyle to the tibial plateau. The joint capsule was dissected to expose the medial compartment, and the medial meniscotibial ligament was transected using a No. 11 microsurgical blade. The anterior horn of the medial meniscus was partially resected along the medial collateral ligament under direct visualization, ensuring tibial cartilage integrity was preserved. Joint instability was confirmed through manual testing, followed by layered closure of the capsule and skin using 5-0 absorbable sutures. Postoperative penicillin (40,000 IU/kg) was administered intramuscularly for 3 days to prevent infection.

At the experimental endpoints, animals were first deeply anesthetized with isoflurane inhalation (5% induction, 1.5%–2% maintenance) using a small animal gas anesthesia system. Once deep anesthesia was confirmed, euthanasia was performed by CO_2_ inhalation using a gradual-fill method at a flow rate of approximately 20%–30% of the chamber volume per minute, followed by confirmation of death. All efforts were made to minimize animal suffering and to reduce the number of animals used.

### Intra-articular lavage and MPCs injection procedures

Under isoflurane anesthesia, rats were positioned in a flexed-knee posture, and the injection area was shaved and disinfected with 10% povidone-iodine (three cycles). A 27-gauge needle was inserted medial to the patellar tendon into the knee joint cavity, and entry was confirmed by the loss of resistance and passive joint movement. For the Joint Lavage and Joint Lavage + MPCs groups, 150 μL of sterile PBS was injected and aspirated through three cycles to complete lavage. After the procedure, the needle was removed, and the skin was re-disinfected.

**FIGURE 1:**
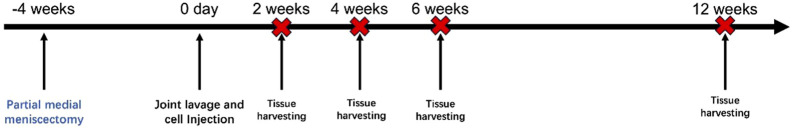
Animal experimental design and workflow. Animal experimental flowchart.

For cell therapy, the MPCs and Lavage + MPCs groups received 100 μL of MPCs suspension (1 × 10^6^ cells/100 μL), while the Sham, Untreated and Lavage groups received 100 μL PBS. Both lavage and cell injection procedures were performed on the same day. The experimental timeline was outlined in Figure 1.

### Quantification of inflammatory cytokines

Lavage fluid from the knee joints was analyzed for inflammatory cytokine levels using Luminex liquid-phase suspension array technology (Wayen Biotechnologies, China). Notably, Due to the limited volume of the rat joint cavity, native synovial fluid could not be obtained in sufficient quantity for reliable cytokine analysis. Therefore, cytokine levels in all groups were measured using joint lavage fluid, which was collected by injecting 100 μL PBS into the joint cavity followed by complete aspiration. Following the manufacturer’s protocol, lavage fluid samples were incubated for 1 h in 96-well plates pre-coated with microspheres from the Bio-Plex Pro Rat Cytokine 23-plex Panel (Grp I; Bio-Rad, USA). After washing, detection antibodies were added and incubated for 30 min, followed by incubation with streptavidin-PE conjugate (10 min). Fluorescence signals were measured using a Bio-Plex MAGPIX system (Bio-Rad, USA), and cytokine concentrations were quantified using standard curves. All datasets were globally standardized using z-scores prior to intergroup statistical analysis. The full raw Lavage fluid Luminex data was provided in Supplementary Table S2.

### Macroscopic analysis

After euthanasia, the knee joints were dissected, and adjacent soft tissues were removed to expose the tibial plateau and femoral condyle. Articular cartilage surfaces were stained with 0.1% India ink (Sigma-Aldrich, USA) for 1 min using a cotton swab. Excess ink was gently removed using PBS-moistened swabs to assess surface integrity. The medial meniscus was carefully isolated under direct visualization. All specimens were photographed under standardized bright-field illumination using a Nikon D850 digital camera (Japan). Macroscopic evaluation of the femoral condyle and tibial plateau cartilage was performed using the International Cartilage Repair Society (ICRS) cartilage lesion classification criteria (Brittberg and Winalski, 2003).

### Histological analysis

Tibial plateau and meniscal tissues were fixed in 4% paraformaldehyde for 48 h, decalcified in EDTA (pH 7.2, Biosharp, China) for 6 weeks, and embedded in paraffin. Tibial plateau specimens were sectioned coronally (3 μm thick), while menisci were sectioned horizontally. Sections were stained with hematoxylin/eosin (HE) and Safranin O (S-O) for proteoglycan visualization. Cartilage degeneration was graded using the Osteoarthritis Research Society International (OARSI) grading system (Pritzker et al., 2006) and the modified Mankin scoring system (Mankin et al., 1971). Meniscal histological evaluation was performed using the modified Pauli’s scoring system (Okuno et al., 2014; Pauli et al., 2012).

For immunohistochemistry, sections were baked at 60 °C for 2 h, deparaffinized, and hydrated with gradient ethanol, followed by antigen retrieval using pepsin (37 °C, 30 min). Endogenous peroxidase activity was blocked with 3% hydrogen peroxide, followed by blocking with 5% BSA (37 °C, 1 h). Primary antibodies against COL1 (1:500, Proteintech, USA) and COL2A1 (1:200, OriGene, USA) were applied overnight at 4 °C. After PBST washes, sections were incubated with horseradish peroxidase (HRP)-conjugated goat anti-rabbit IgG secondary antibody (37 °C, 1 h), developed with 3,3′-diaminobenzidine (DAB), counterstained with hematoxylin, dehydrated, and mounted. The medial region of the regenerated meniscus was selected as the region of interest because it best reflects the collagen structure and quality of the repair. For each group and each time point, sections from four rats were evaluated. Two consecutive sections were used for COL I and COL II staining, and the same representative area was consistently selected for analysis to ensure consistency across samples.

All slides were digitally scanned using a Wanfu Slide Scanner (PANNORAMIC 250, China) under bright-field illumination. Quantitative analysis of the staining area ratio was performed using ImageJ software (NIH, USA).

### Biomechanical properties evaluation

A randomly selected medial meniscus from each group at the 12-week timepoint underwent tensile test to assess biomechanical properties. The posterior horn of the meniscus was clamped laterally with a hemostat, while the distal end of the regenerated tissue was secured with a second hemostat. Specimens were mounted on a testing machine (Testometric AX, M350-10kN, United Kingdom) and preconditioned with a 2 N preload. Load-to-failure testing was conducted at a rate of 1 mm/s. The failure load was defined as the maximum force before tissue rupture. Stiffness was calculated as the slope of the linear region of the load-displacement curve using Origin software (OriginLab, USA).

### Statistical analysis

Statistical analyses were conducted using GraphPad Prism (v9.3.0, GraphPad Software, San Diego, CA, USA). Intergroup differences were analyzed using unpaired Student’s t-tests. Pairwise comparisons between multiple groups were analyzed using one-way or two-way ANOVA, depending on the number of experimental variables. Data were presented as the mean ± standard deviation (SD) in both figures and text. Statistical significance was set at P < 0.05.

## Results

### Joint lavage reduces concentrations of IL-1β and multiple cytokines in rat models

Analysis of inflammatory cytokine concentrations in lavage fluid across timepoints revealed significantly elevated levels of IL-1β and IL-18 in the non-lavage group (0 days) compare to sham group (P < 0.001; Supplementary Figure S1A). This result indicated that the surgical model effectively induced inflammatory environment of the knee joint. The joint lavage significantly reduced the levels of multiple cytokines (IL-1β, IL-17A, MCP-1, IL-18) at 2, 4, and 6 weeks (P < 0.0001; Supplementary Figure S1A). This suppression pattern was further corroborated by heatmap analysis (Supplementary Figure S1B). Collectively, these data indicated that joint lavage provided sustained suppression of pro-inflammatory cytokines.

### Assessment of MPCs viability, proliferation and clonogenic capacity

**FIGURE 2:**
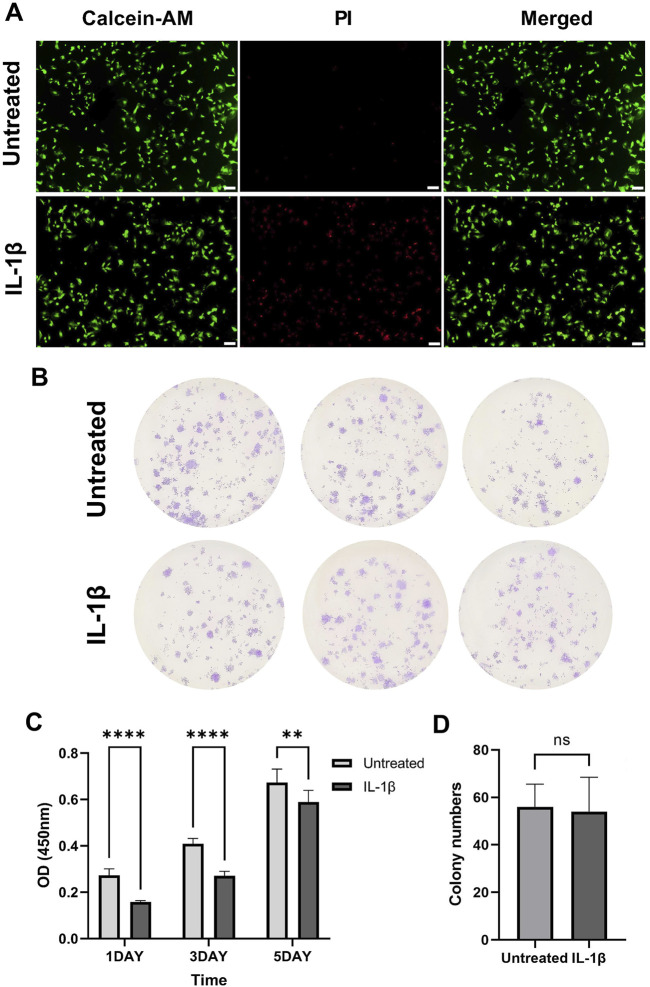
IL-1β impairs the viability and proliferative capacity of meniscus progenitor cells (MPCs). **(A)** Representative live/dead staining images of untreated and IL-1β-treated MPCs, showing increased cell death following IL-1β exposure. **(B)** Representative images of colony-forming unit assay. **(C)** CCK-8 proliferation assay demonstrates significantly reduced cell proliferation in IL-1β-treated MPCs. **(D)** Colony numbers showed no significant difference between the two groups. Scale bar: 200 μm. ns: not significant, **P < 0.01, ****P < 0.0001.

To study the effect of the inflammatory environment on the properties and function of MPCs, we exposed isolated MPCs to IL-1β *in vitro* for 24 h. Live/dead staining revealed a significant increase in PI-positive dead cells in IL-1β-treated MPCs (IL-1β) compared to controls (Untreated) after 24-h treatment (Figure 2A). This result indicated that IL-1β suppressed cell viability. CCK-8 proliferation assays showed that IL-1β treatment significantly inhibited MPCs short-term proliferative activity (P < 0.01; Figure 2C). In contrast, colony formation analysis revealed no significant differences in colony numbers between two groups (ns: not significant; Figures 2B,D). This implied that IL-1β did not significantly affect the long-term proliferative potential of MPCs.

### Multilineage differentiation potential and cell migration

**FIGURE 3:**
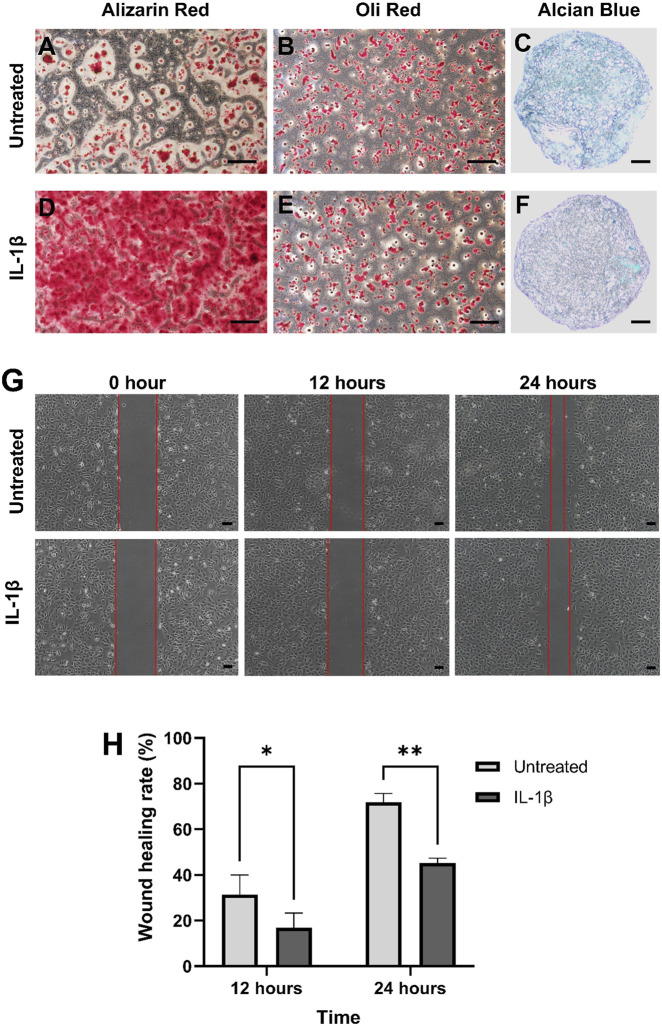
IL-1β suppresses the chondrogenic differentiation and migration of MPCs. Untreated and IL-1β-treated MPCs were subjected to **(A,D)** osteogenic, **(B,E)** adipogenic, and **(C,F)** chondrogenic differentiation assays. IL-1β enhanced osteogenic differentiation but markedly inhibited chondrogenic matrix formation. **(G)** Representative scratch assay images showing reduced cell migration following IL-1β treatment. **(H)** Quantification of wound healing rates confirms significantly impaired migratory capacity. Scale bar: 200 μm. *P < 0.05, **P < 0.01.

IL-1β-treated MPCs showed wider stained surface area of Alizarin Red compared to controls following osteogenic induction (Figures 3A,D), indicating enhanced osteogenic differentiation. However, no significant differences in Oil Red O staining were observed between the two groups following adipogenic induction (Figures 3B,E). Chondrogenic cultures revealed that IL-1β-treated pellets had sparse Alcian blue staining, whereas control pellets exhibited nearly complete matrix coverage (Figures 3C,F). This indicated that IL-1β suppressed the chondrogenic capacity of MPCs *in vitro*. Additionally, scratch assays revealed significantly slower migration rates in IL-1β-treated MPCs at 12 (P < 0.05) and 24 h (P < 0.01; Figures 3G,H). This confirmed that IL-1β impaired migratory capacity in MPCs.

### Surface marker profiling and gene expression analysis

**FIGURE 4:**
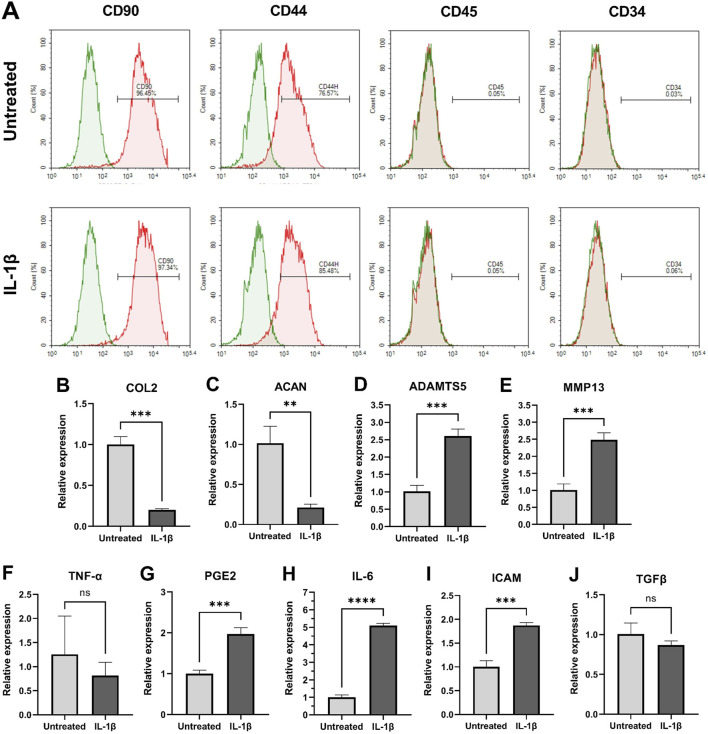
IL-1β alters the gene expression profile of MPCs. **(A)** Flow cytometry analysis shows representative expression patterns of stem/progenitor surface markers in MPCs. Quantitative real-time PCR reveals significant downregulation of chondrogenic genes **(B–E)** and upregulation of inflammatory genes **(F–J)** following IL-1β exposure. ns: not significant, **P < 0.01, ***P < 0.001, ****P < 0.0001.

Flow cytometry analysis showed that MPCs in both groups were positive for CD90 and CD44, while exhibiting low expression of CD45 and CD34 (Figure 4A). The expression patterns of these surface markers were comparable between controls and IL-1β-treated MPCs. qPCR results showed that the expression of COL2 and ACAN in MPCs was significantly downregulated (P < 0.01; Figures 4B,C), coupled with upregulation of ADAMTS5 and MMP13 (P < 0.001; Figures 4D,E) in IL-1β-treated MPCs. These results suggested that IL-1β-mediated suppression of chondrogenic capacity. In addition, IL-1β treatment significantly increased the expression of IL-6 (P < 0.0001; Figure 4H), PGE2, and ICAM (P < 0.001; Figures 4G,I) in MPCs, whereas anti-inflammatory TGF-β remained unchanged (ns: not significant, Figure 4J). TNF-α expression showed no statistical significance due to excessive data variability (Figure 4F).

### Evaluation of regenerated meniscus

**FIGURE 5:**
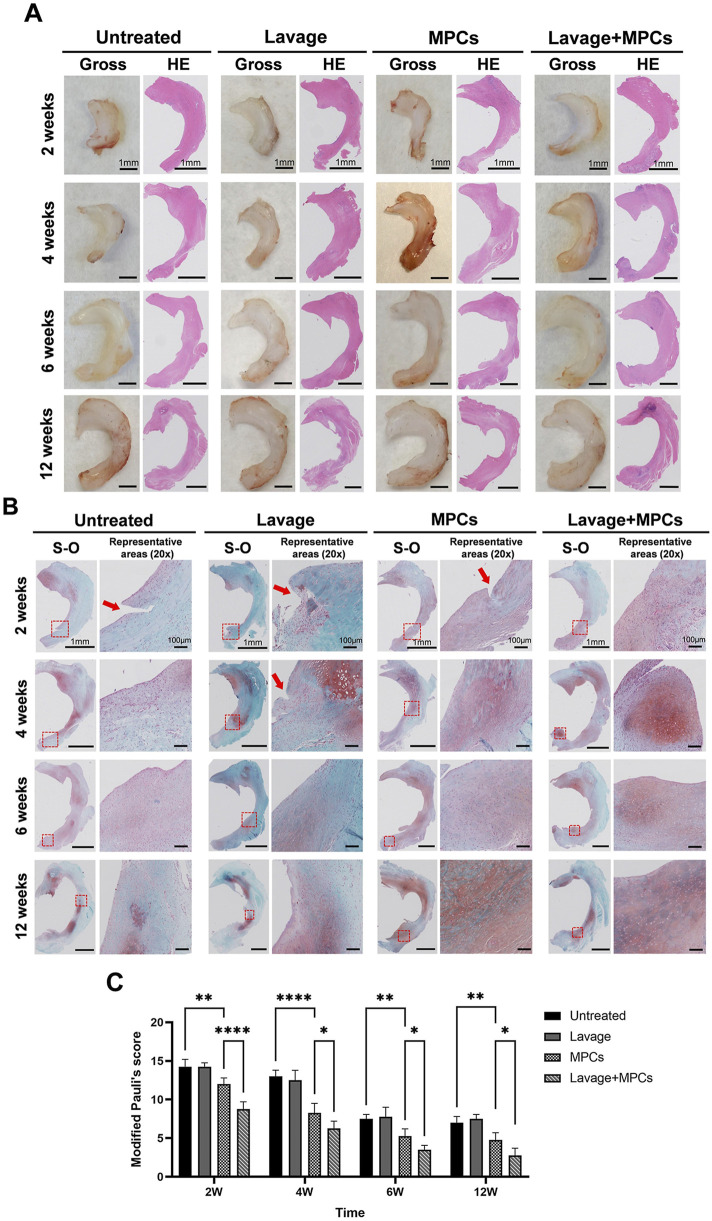
Combined joint lavage and MPC therapy enhances meniscal regeneration in rat models. Macroscopic and histological images of meniscal tissues show improved structural restoration following combined lavage and MPC treatment. **(A)** Hematoxylin and eosin (HE) staining and **(B)** Safranin O–fast green (S–O) staining reveal enhanced tissue integration and matrix deposition compared with other groups. Red arrows indicate unhealed clefts. **(C)** Meniscal histological scores further demonstrate the superior regenerative outcomes in the lavage + MPCs group (n = 4 per group). *P < 0.05, **P < 0.01, ****P < 0.0001.

Representative H&E and Safranin-O stained sections of the medial meniscus for all groups were shown in Figure 5. Progressive meniscal regeneration and enhanced Safranin-O staining were observed across the groups over time. At week 2, an unhealed cleft was evident in the untreated and MPCs groups, whereas the lavage + MPCs group demonstrated seamless tissue integration (Figure 5A). Furthermore, during the early stages, both MPCs and lavage + MPCs groups exhibited greater proteoglycan matrix deposition, particularly within the regenerated tissue near the resection margin where densely packed round cells predominated—features that were notably absent in the untreated and lavage groups (Figure 5B). Notably, at week 6 and 12, the lavage + MPCs group displayed meniscal architecture and histochemical properties that more closely resembled the structure of sham group (Supplementary Figure S2) in both gross morphology and histological staining (Figures 5A,B). By contrast, the untreated group exhibited only moderate or slightly positive Safranin-O staining with a loss of chondrocytes, in line with its poorer meniscal histological scores (Figure 5C). These results indicated that MPCs combined joint lavage better promoted meniscus regeneration.

**FIGURE 6:**
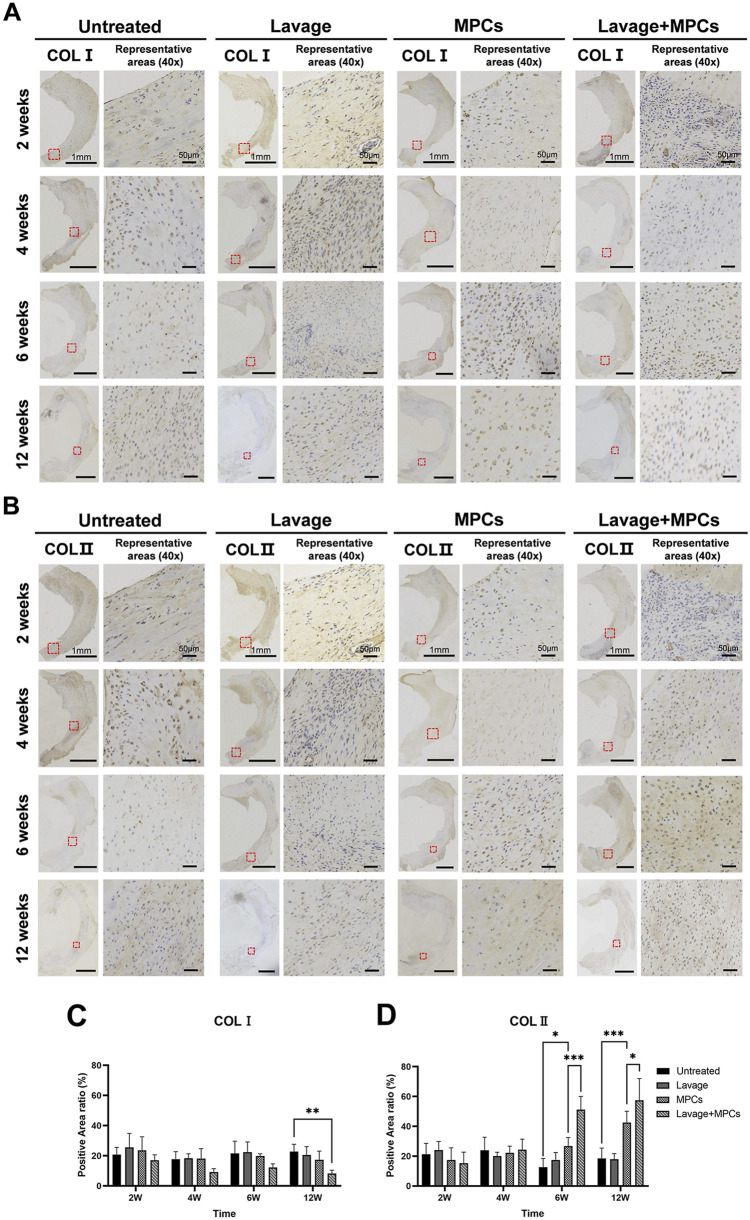
Combined lavage and MPC therapy restores collagen composition in regenerated meniscus. Immunohistochemical staining of regenerated menisci shows distribution patterns of **(A)** type I collagen (COL I) and **(B)** type II collagen (COL II). Semi-quantitative analysis demonstrates significantly decreased COL I and increased COL II deposition in the lavage + MPC group by week 12. **(C,D)** Quantification of COL I and COL II immunostaining (n = 4 per group). *P < 0.05, **P < 0.01, ***P < 0.001.

Immunohistochemical analysis of COL I and COL II distribution within the regenerated meniscus was performed on the consistent representative areas of tissue for quantitative assessment. In sham group, COL I predominantly localized to the peripheral region, while COL II exhibited greater abundance in the inner region, particularly within the anterior horn (Supplementary Figure S3). During the initial phase (week 2 and 4), there were no significant differences in COL I and COL II content among all groups. Although the MPCs group exhibited significantly higher COL II expression compared to untreated group (P < 0.05) from week 6 onward, it remained lower than the lavage + MPCs group (P < 0.001), which reached threefold that of the untreated group at week 12 (Figures 6B,D). Notably, the lavage + MPCs group exhibited marked reduction in COL I staining intensity by week 12 (P < 0.01, Figures 6A,C). These results suggested lavage enhances the role of MPCs in the meniscal regeneration process dominated by COL II formation.

### Biomechanical characterization of the regenerated meniscus

Tensile testing of week 12 meniscus revealed complete tissue rupture under load (Supplementary Figures S4A,B). Force-deformation curves were derived from the experimental data, with tabulated results presented in Supplementary Table S3. Although no significant difference in ultimate load was observed between the MPCs and lavage + MPCs groups, the lavage + MPCs group showed a 2.3-fold increase in stiffness compared to untreated group. Collectively, these findings suggested that MPCs therapy significantly improves the biomechanical integrity of regenerated meniscal tissue, and joint lavage further optimized stiffness properties.

### Assessment of cartilage degeneration

**FIGURE 7:**
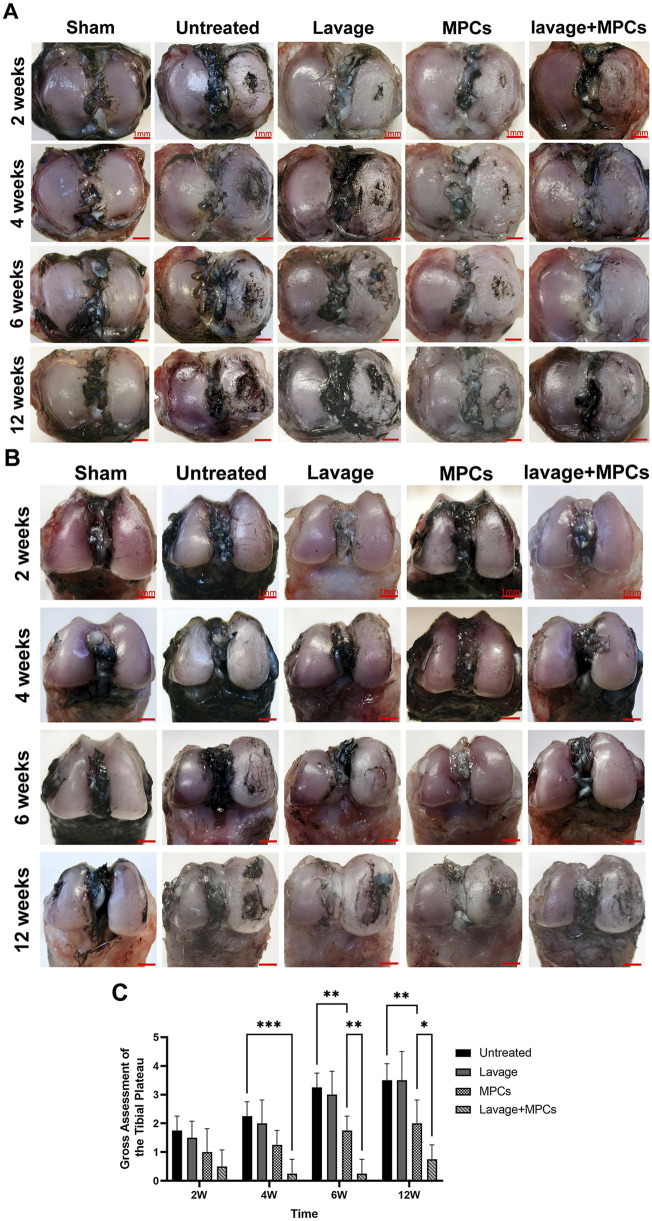
Joint lavage combined with MPC therapy improves articular cartilage morphology. Gross observations of articular cartilage in the **(A)** tibial plateau and **(B)** femoral condyle show reduced lesion severity in the lavage + MPC group. **(C)** Macroscopic scoring demonstrates significant cartilage preservation and reduced degeneration compared with other groups (n = 4 per group). *P < 0.05, **P < 0.01, ***P < 0.001.

Macroscopic India ink staining showed aggravated cartilage lesions on the tibial plateaus in both untreated and lavage groups, with large lesion areas by week 12. While the MPCs group developed significant cartilage damage from week 4 onward, the lavage + MPCs group exhibited only superficial surface fibrillation (Figure 7A). Gross assessment of the joint changes score system (Figure 7C) demonstrated that MPCs treatment delayed tibial plateaus cartilage deterioration from week 6 onward (P < 0.01), whereas the lavage + MPCs group achieved lower pathological scores than MPCs monotherapy (P < 0.01). Femoral condyles cartilage defects became macroscopically evident at week 6 across untreated and lavage groups. In both MPCs and lavage + MPCs groups, lesions were confined to surface fibrillation or minimal cartilage damage (Figure 7B).

**FIGURE 8:**
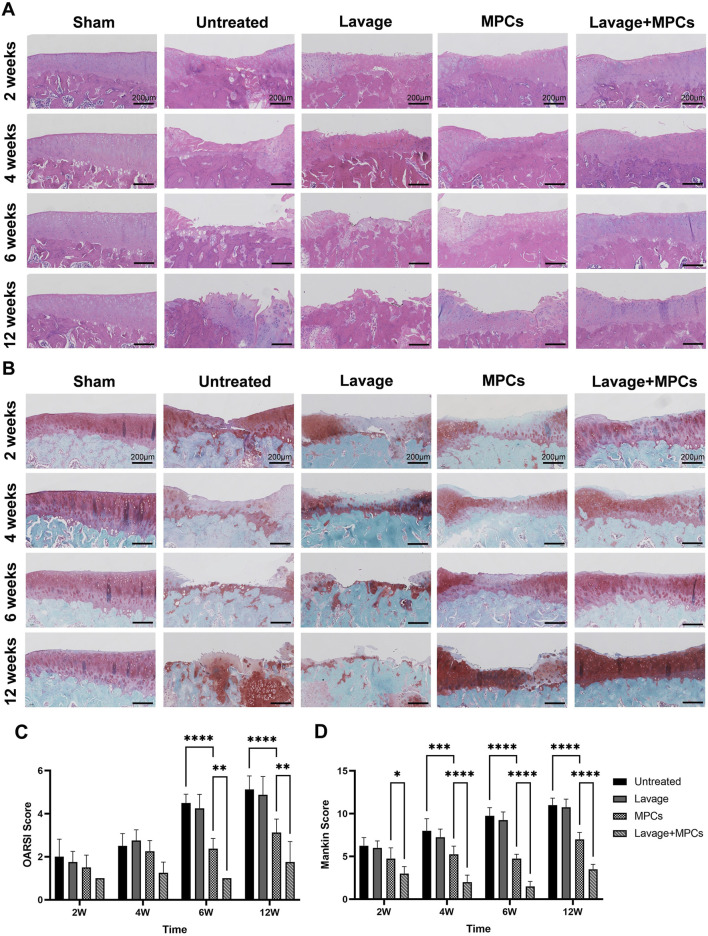
Joint lavage combined with MPC therapy protects articular cartilage from degeneration. **(A)** Hematoxylin and eosin (HE) and **(B)** Safranin O–fast green (S–O) staining of tibial plateau cartilage surfaces show reduced structural damage in the lavage + MPC group. **(C)** Osteoarthritis Research Society International (OARSI) scores and **(D)** modified Mankin scores demonstrate significantly improved cartilage preservation compared with controls (n = 4 per group). *P < 0.05, **P < 0.01, ***P < 0.001, ****P < 0.0001.

Histological analysis revealed progressive exacerbation of tibial plateaus cartilage over time across all groups, most severely in the untreated and lavage groups, which exhibited extensive full-thickness defects and markedly diminished Safranin-O staining from week 6 onward (Figures 8A,B). While isolated MPCs injection retarded cartilage degeneration progression, damage extending to the middle layer of cartilage was observed by week 12. In contrast, the lavage + MPCs group displayed only superficial cartilage fibrillation or disorganization. This group also showed significantly lower OARSI and Mankin scores (P < 0.0001; Figures 8C,D).

## Discussion

Stem cell therapy is widely recognized as a promising approach for promoting meniscal tissue regeneration; however, the inflammatory environment within the knee joint limits its therapeutic efficacy (Peng et al., 2022). In this study, we established a meniscectomy rat model and evaluated the effect of joint lavage on MPCs regenerative efficacy. Additionally, IL-1β treatment was used to induce a pro-inflammatory environment *in vitro*. We found that joint lavage could effectively reduce the concentrations of various pro-inflammatory cytokines. Our *in vivo* findings showed that joint lavage prior to MPCs injection effectively suppressed IL-1β- dominated inflammatory cytokines, allowing MPCs to fully activate their regenerative potential and achieve superior structural and functional restoration within the joint.

*In vitro*, IL-1β suppressed the viability, proliferation, migration, and chondrogenic differentiation of MPCs. This likely explained the limited repair observed following MPCs monotherapy: injected MPCs may become dysfunctional when exposed to the inflammatory environment following implantation. Our *in vivo* data revealed that MPCs group resulted in only partial meniscal regeneration and limited chondroprotection. Although this group exhibited increased safranin-O staining and type II collagen deposition compared to the untreated group, the results were far inferior to those of sham group. Notably, significant cartilage defects emerged on the tibial plateau and femoral condyles in later stages.

Previous studies have shown that in surgically induced rat osteoarthritis models, multiple pro-inflammatory cytokines in the synovial fluid remain elevated for prolonged periods (8–10 weeks) without spontaneous resolution (Wang et al., 2020; Busa et al., 2022; Allen et al., 2020; Ferrándiz et al., 2014). In contrast, Repeated joint lavage reduced intra-articular cytokines, including IL-1β, helping to improve the inflammatory environment. When combined with lavage, MPCs transplantation produced regenerated meniscus with better histological scores, higher type II collagen deposition, and superior mechanical stiffness. Notably, protection of articular cartilage was also observed in lavage + MPCs group. These findings highlight that controlling inflammation environment—particularly by suppressing IL-1β—is crucial for MPCs efficacy, validating the necessity of pre-treatment joint lavage. And this approach features low cost and procedural simplicity.

A clinical study reported favorable outcomes with joint lavage combined with stem cell injection (Koh et al., 2015); however, these results were often attributed primarily to stem cell therapy rather than lavage. We also conclude that isolated joint lavage is insufficient for effective meniscal regeneration or preventing cartilage damage, as evidenced by our lavage group in animal experiments. Although lavage alone may not provide long-term therapeutic benefits, it creates an optimal microenvironment for subsequent MPCs therapy. However, it is important to note that the effectiveness of lavage is closely related to the physicochemical properties of the irrigation solution. Several studies have shown that hyperosmotic and appropriately warmed solutions can reduce chondrocyte death, enhance tissue resilience, and help preserve matrix integrity, suggesting that optimized lavage conditions may offer chondroprotective benefits (Eltawil et al., 2018; Eltawil et al., 2015; Amin et al., 2008; Unterguggenberger et al., 2024). In this study, only a single lavage protocol was used (PBS solution, 22 °C, applied three consecutive times). Thus, our results represented only the lavage conditions used in this study and could not rule out the potential benefits of other lavage protocols.

A rat-based study suggested that days 7–14 post-injury may represent the optimal therapeutic window for stem cell therapy (Zhao et al., 2025); similarly, most clinical trials administered MSCs injections within 2 weeks after meniscal injury or meniscectomy, yielding favorable outcomes (Sekiya et al., 2019; Vangsness et al., 2014). However, in clinical practice, patients usually consider cell-based therapies only after developing symptoms such as pain or functional impairment, often beyond this early phase. This prompted us to select a 4-week postoperative interval for intervention, when rat models exhibited early signs of PTOA (Furuoka et al., 2023; Yanagisawa et al., 2016), better reflecting common clinical scenarios. Our results showed that a single MPCs injection had limited effects on meniscus regeneration and cartilage protection. However, joint lavage before injection significantly enhanced therapeutic efficacy of MPCs. These findings suggest that our combination strategy may be a feasible option even for patients who have missed the optimal therapeutic window, thereby expanding its clinical applicability and reinforcing its translational potential.

Notably, although lavage + MPCs proved effective, the regenerated meniscal tissue still differed from native meniscus, and surface fibrillation was observed on the tibial plateau and medial femoral condyle in the later stages post-treatment. This may be because, although joint lavage effectively reduces intra-articular inflammatory cytokines long enough for MPCs to exert their regenerative function, structural damage—including cartilage degeneration and meniscal injury—had already developed by 4 weeks post-surgery. In addition, the abnormal joint biomechanics caused by partial meniscectomy cannot be fully restored by lavage alone. This limitation ultimately hinders repair and regeneration outcomes. Future studies may explore strategies that combine inflammation modulation with biomechanical restoration to enhance MPCs-mediated meniscus regeneration and cartilage protection.

This study has several limitations. First, due to ethical constraints, rats and rat MPCs were used for experiment. While ensuring species consistency and preventing xenotransplantation rejection (Pei et al., 2010), rodent findings may not fully reflect human MPCs behavior and function, lacking validation in human models. Moreover, only male animals were used to avoid sex-based differences, which may limit the generalizability of the findings. In addition, rats possess a certain innate capacity for meniscal self-healing, which may limit how well this model reflects human meniscal injury and repair and affect the perceived treatment efficacy (Yan et al., 2024a; Shen et al., 2014). Consequently, future investigations will employ physiologically and anatomically relevant animal models (such as sheep, goats, and non-human primates) to evaluate the therapeutic potential of MPCs in cartilage and meniscal repair, thereby accelerating clinical translation. Second, our *in vitro* model relied solely on IL-1β, which failed to replicate the intricate *in vivo* inflammatory environment characterized by cascading interactions involving multiple cytokines, inflammatory mediators, and immune cells. Subsequent studies may examine additional inflammatory factors and mediators. Third, while phenotypic assessments were performed, the molecular mechanisms remain undefined; future research should prioritize elucidating specific cellular signaling pathways, which are essential for developing effective regenerative therapies. Despite these limitations, our protocol provides a valuable reference framework for MPCs applications.

## Data Availability

The original contributions presented in the study are included in the article/Supplementary Material, further inquiries can be directed to the corresponding authors.
