# Supplementary material for: Meniscus progenitor cells combined with joint lavage promote meniscus regeneration and cartilage protection in rat models
Source: Front Bioeng Biotechnol. 2026 Jan 21;13:1724656. doi: 10.3389/fbioe.2025.1724656 (PMC12884168; doi:10.3389/fbioe.2025.1724656)
Supplement: Supplementary file 1 [file Supplementaryfile1.docx]

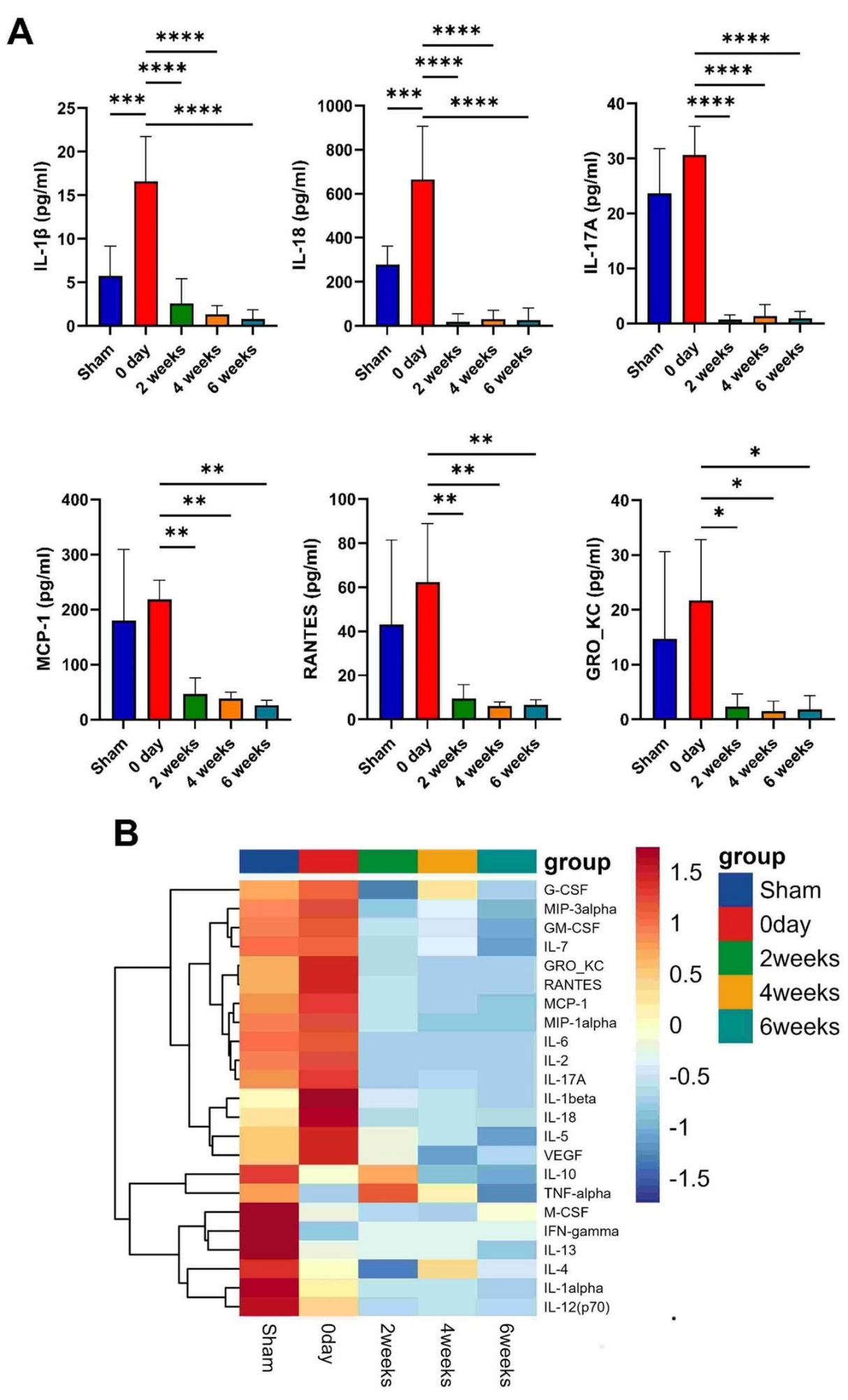


Figure S1. Inflammatory cytokine profiling in lavage fluid. Assessment of inflammatory cytokines in lavage fluid. (A) Levels of pro-inflammatory cytokines such as IL-1β. (B) Heatmap of the relative expression of inflammatory cytokines. Columns represent sample groups, and rows represent cytokine species. *P < 0.05, **P < 0.01, ***P < 0.001, ****P < 0.0001.


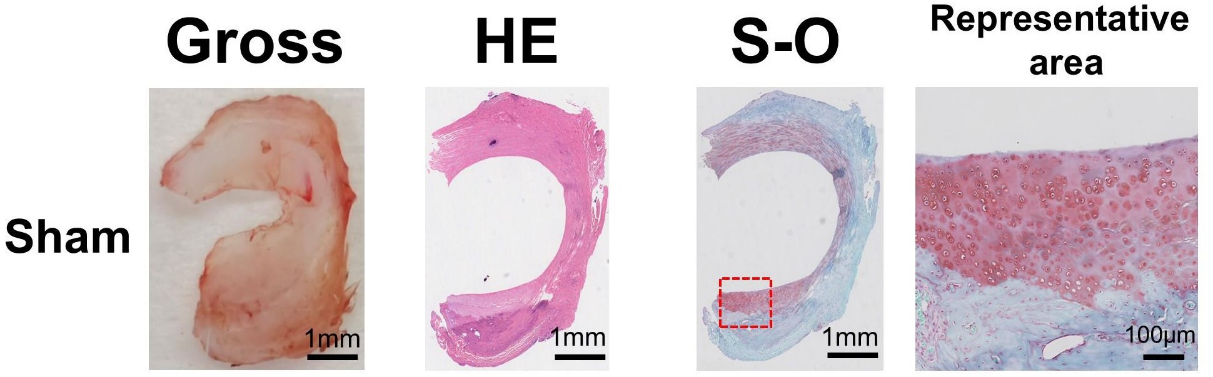


Figure S2. Baseline meniscal morphology in sham group. Macroscopic, Hematoxylin and eosin (HE) staining and Safranin O-fast green (S-O) staining images (general view and representation area) of menisci in sham group.


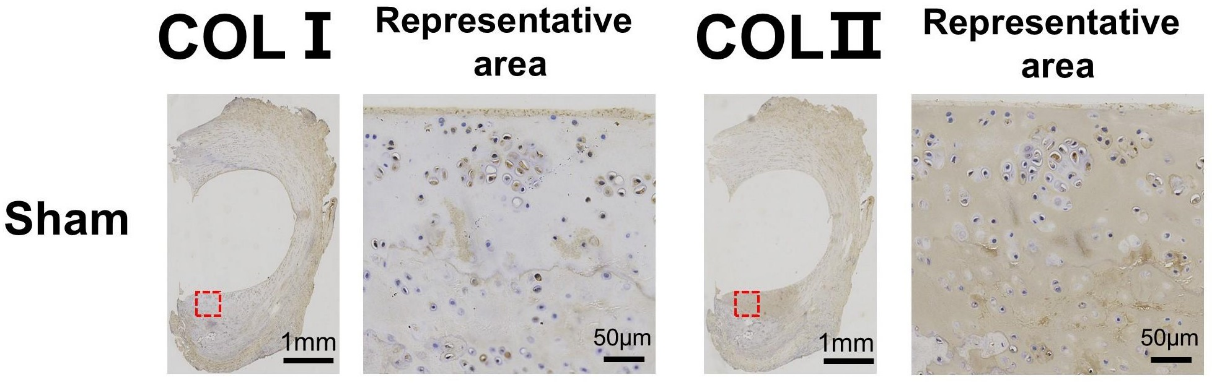


Figure S3. Collagen composition of menisci in sham group. Immunohistochemical staining for type I collagen (COL I) and type II collagen (COL II) images (general view and representation area) of menisci in sham group.


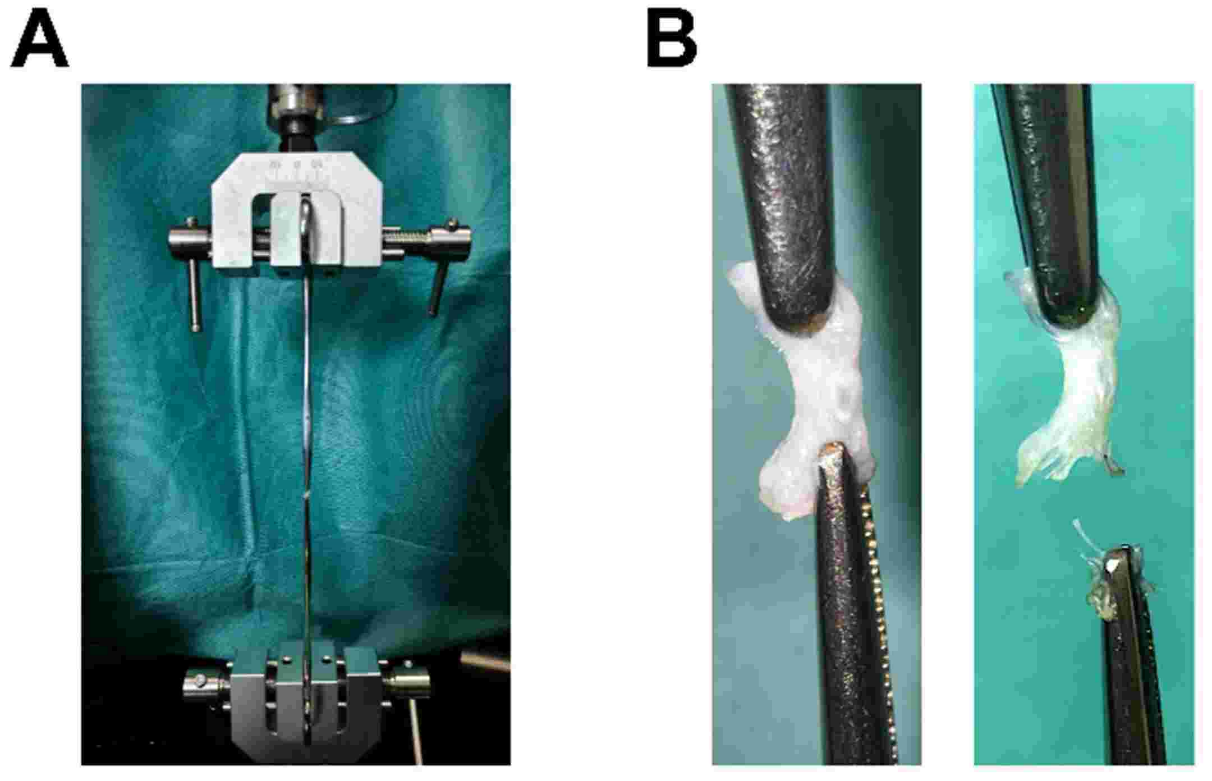


Figure S4. Biomechanical testing of meniscal specimens. Representative images showing specimen fixation (A) and tensile loading to failure (B) used to evaluate meniscal mechanical properties.

| **Gene (Rat)** | **Forward primer (5'-3')** | **Reverse primer (5'-3')** |
| --- | --- | --- |
| **MMP-13** | ACCCTGGAGCCCTGATGTTT | GTAATGGCATCAAGGGATAGGG |
| **COL2a1** | ACCGTGGTGACAAGGGAGAGA | ACCACCAGCCTTCTCGTCAA |
| **ACAN** | AGTGACCCATCTGCTTACCCTG | CTGCATCTATGTCGGAGGTAGTG |
| **TNF-α** | CCACCACGCTCTTCTGTCTACTG | TGGGCTACGGGCTTGTCACT |
| **ICAM-1** | CTCTTGCGAAGACGAGAACCTC | CTCGCTCTGGGAACGAATACAC |
| **IL-6** | AGCTATGAAGTTTCTCTCCGCAAG | ATACTGGTCTGTTGTGGGTGGT |
| **TGF-β** | GCTGAACCAAGGAGACGGAATA | GCAGGTGTTGAGCCCTTTCC |
| **ADANTS5** | ACAAGAGTCTGGAGGTGAGCAAG | ACATATGGTCCCAACGTCTGC |
| **PGE2** | TGACTGTACCCGGACTGGAT | TCCCTTGAAGTGGGTCAGGA |
| **GAPDH** | CTGGAGAAACCTGCCAAGTATG | GGTGGAAGAATGGGAGTTGCT |

Table S1. Primer sequences used for RT-qPCR. Forward and reverse primer sequences for chondrogenic and pro-inflammatory genes analyzed by RT-qPCR. GAPDH is a housekeeping gene.

| **Name** | **G-CSF** | **GM-CSF** | **GRO_KC** | **IFN-γ** | **IL-1α** | **IL-1β** | **IL-2** | **IL-4** | **IL-5** | **IL-6** | **IL-7** | **IL-10** | **IL-12** | **IL-13** | **IL-17A** | **IL-18** | **M-CSF** | **MCP-1** | **MIP-1α** | **MIP-3α** | **RANTES** | **TNF-α** | **VEGF** |
| --- | --- | --- | --- | --- | --- | --- | --- | --- | --- | --- | --- | --- | --- | --- | --- | --- | --- | --- | --- | --- | --- | --- | --- |
| **Sham1** | 0.03 | 3.91 | 15.18 | 0.005 | 12.68 | 6.53 | 73.15 | 5.93 | 34.2 | 7.35 | 2.17 | 2.59 | 12.55 | 2.9 | 29.27 | 211.51 | 0.98 | 307.24 | 2.98 | 0.78 | 56.97 | 17.17 | 84.9 |
| **Sham2** | 0.03 | 3.11 | 37.09 | 3.48 | 87.17 | 10.26 | 73.15 | 0.95 | 38.92 | 7.35 | 1.36 | 6.44 | 5.13 | 2.9 | 32.07 | 281.2 | 0.44 | 275.29 | 3.35 | 1.22 | 91.16 | 17.17 | 91.6 |
| **Sham3** | 0.21 | 3.37 | 3.1 | 15.11 | 35.65 | 3.12 | 146.3 | 3.35 | 43.31 | 14.7 | 0.82 | 6.44 | 36.03 | 36.15 | 16.71 | 224.04 | 0.98 | 65.61 | 0.265 | 0.93 | 11.95 | 53.79 | 17.04 |
| **Sham4** | 0.21 | 3.37 | 3.64 | 15.11 | 31.14 | 3.12 | 146.3 | 3.35 | 43.31 | 7.35 | 1.36 | 2.59 | 12.55 | 11.14 | 16.71 | 395.55 | 0.44 | 70.82 | 0.53 | 0.78 | 12.38 | 33.64 | 10.28 |
| **0day-1** | 0.17 | 3.91 | 8.92 | 0.005 | 31.14 | 11.01 | 73.15 | 3.35 | 51.36 | 14.7 | 1.09 | 1.295 | 20.22 | 11.14 | 36.54 | 429.21 | 0.22 | 181.82 | 2.79 | 0.71 | 31.71 | 40.76 | 47.63 |
| **0day-2** | 0.21 | 3.91 | 27.07 | 0.01 | 11.01 | 17.57 | 244.6 | 3.35 | 58.7 | 7.35 | 2.44 | 6.44 | 5.13 | 2.9 | 26.78 | 910.83 | 0.22 | 252.28 | 3.35 | 1.51 | 77.49 | 8.585 | 81.83 |
| **0day-3** | 0.03 | 3.37 | 29.13 | 0.005 | 13.23 | 21.15 | 73.15 | 0.95 | 23.24 | 7.35 | 0.82 | 1.295 | 2.565 | 2.9 | 28.56 | 656.49 | 0.22 | 220.76 | 0.265 | 0.93 | 78.02 | 8.585 | 79.6 |
| **2week1** | 0.05 | 1.11 | 2.03 | 1.74 | 5.22 | 0.97 | 6.535 | 0.95 | 51.36 | 2.295 | 0.075 | 3.95 | 2.17 | 2.76 | 0.14 | 1.89 | 0.075 | 20.48 | 0.005 | 0.14 | 3.05 | 67.07 | 22.06 |
| **2week2** | 0.025 | 2.08 | 4.68 | 1.74 | 24.68 | 4.57 | 6.535 | 2.12 | 34.2 | 2.295 | 1.23 | 6.34 | 2.17 | 2.76 | 1.61 | 85.2 | 0.15 | 71.48 | 1.01 | 0.54 | 13 | 37.62 | 52.34 |
| **2week3** | 0.025 | 0.44 | 0.205 | 1.74 | 0.31 | 0.73 | 6.535 | 0.475 | 34.2 | 2.295 | 0.075 | 1.41 | 2.17 | 2.76 | 0.14 | 1.89 | 0.075 | 28.67 | 0.005 | 0.14 | 4.88 | 8.16 | 30.61 |
| **2week4** | 0.05 | 2.84 | 4.68 | 1.74 | 9.07 | 6.53 | 6.535 | 4.62 | 29.04 | 2.295 | 0.69 | 6.34 | 2.17 | 15.16 | 1.61 | 1.89 | 0.15 | 84.74 | 0.44 | 0.67 | 18.85 | 16.32 | 62.24 |
| **2week5** | 0.025 | 0.66 | 0.205 | 1.74 | 1.39 | 0.02 | 6.535 | 0.475 | 34.2 | 2.295 | 0.075 | 1.41 | 2.17 | 2.76 | 0.14 | 1.89 | 0.075 | 27.31 | 0.005 | 0.03 | 7.49 | 37.62 | 21 |
| **4week1** | 0.14 | 1.59 | 0.96 | 1.74 | 6.32 | 1.2 | 6.535 | 2.12 | 23.24 | 2.295 | 0.42 | 3.95 | 2.17 | 15.16 | 0.14 | 62.01 | 0.075 | 42.83 | 0.005 | 0.4 | 3.56 | 8.16 | 10.03 |
| **4week2** | 0.05 | 0.44 | 0.205 | 1.74 | 5.76 | 1.2 | 6.535 | 2.12 | 23.24 | 2.295 | 0.075 | 1.41 | 2.17 | 2.76 | 1.61 | 1.89 | 0.075 | 45.51 | 0.005 | 0.54 | 8.34 | 16.32 | 36 |
| **4week3** | 0.14 | 3.63 | 4.68 | 1.74 | 17.97 | 2.64 | 6.535 | 4.62 | 58.7 | 2.295 | 1.5 | 3.95 | 4.34 | 2.76 | 4.78 | 85.2 | 0.15 | 48.85 | 0.01 | 0.67 | 7.88 | 67.07 | 27.12 |
| **4week4** | 0.025 | 0.24 | 0.205 | 1.74 | 5.76 | 0.04 | 6.535 | 0.475 | 34.2 | 2.295 | 0.075 | 0.705 | 2.17 | 2.76 | 0.14 | 1.89 | 0.075 | 21.86 | 0.005 | 0.14 | 5.69 | 8.16 | 8.49 |
| **4week5** | 0.14 | 1.83 | 1.49 | 1.74 | 3.57 | 1.68 | 6.535 | 4.62 | 34.2 | 2.295 | 0.96 | 0.705 | 2.17 | 2.76 | 0.14 | 1.89 | 0.075 | 32.73 | 0.005 | 0.54 | 4.72 | 28.14 | 16.79 |
| **6week1** | 0.025 | 0.66 | 6.27 | 1.74 | 11.29 | 0.02 | 6.535 | 2.12 | 34.2 | 2.295 | 0.075 | 0.705 | 2.17 | 2.76 | 0.14 | 1.89 | 0.075 | 21.86 | 0.005 | 0.14 | 4.88 | 16.32 | 30.34 |
| **6week2** | 0.14 | 2.08 | 0.205 | 1.74 | 1.39 | 1.68 | 6.535 | 4.62 | 34.2 | 2.295 | 0.42 | 6.34 | 2.17 | 5.52 | 1.61 | 1.89 | 0.92 | 28.67 | 0.005 | 0.27 | 6.01 | 8.16 | 13.65 |
| **6week3** | 0.025 | 0.03 | 0.205 | 1.74 | 0.155 | 0.02 | 6.535 | 2.12 | 23.24 | 2.295 | 0.075 | 0.705 | 2.17 | 2.76 | 0.14 | 1.89 | 0.075 | 16.35 | 0.005 | 0.14 | 6.01 | 8.16 | 30.07 |
| **6week4** | 0.05 | 0.06 | 1.49 | 1.74 | 1.39 | 0.02 | 6.535 | 0.475 | 34.2 | 2.295 | 0.075 | 0.705 | 2.17 | 2.76 | 0.14 | 1.89 | 0.075 | 23.22 | 0.005 | 0.03 | 5.85 | 8.16 | 34.11 |
| **6week5** | 0.05 | 1.11 | 0.96 | 1.74 | 8.52 | 2.16 | 6.535 | 2.12 | 34.2 | 2.295 | 0.15 | 1.41 | 2.17 | 2.76 | 2.74 | 123 | 0.075 | 40.82 | 0.005 | 0.67 | 10.59 | 37.62 | 31.14 |

Table S2. Raw synovial fluid Luminex data. Concentrations (pg/mL) of all measured cytokines from each individual sample.

|  | **Slope (representing stiffness)** | **Maximum Load (unit: N)** |
| --- | --- | --- |
| **Sham** | 62.13475 | 29.7955 |
| **Untreated** | 17.85462 | 10.371 |
| **Lavage** | 28.0651 | 11.216 |
| **MPCs** | 32.67691 | 20.49 |
| **Lavage + MPCs** | 40.74976 | 18.714 |

Table S3. Biomechanical properties of meniscal tissue. Comparison of stiffness and maximum load across sham, untreated, lavage, MPCs and lavage + MPCs groups.
